# Breast tumor with giant borderline phyllodes: Case report and literature review

**DOI:** 10.1097/MD.0000000000037260

**Published:** 2024-11-01

**Authors:** Gongyin Zhang, Jinsheng Zeng, Changwang Li, Changlong Wei

**Affiliations:** a Department of Breast Surgery, The First Affiliated Hospital of Nanchang University, Jiangxi Province, China.

**Keywords:** breast, case report, characteristics, giant borderline phyllodes tumors, treatment

## Abstract

**Rationale:**

Giant phyllodes tumors are rare fibroepithelial neoplasms, accounting for less than 1% of all primary breast tumors. Their main features are a single-round mass, progressive enlargement, and a high rate of local recurrence. A phyllodes tumor measuring more than 10 cm in diameter is usually defined as a “giant” tumor. Surgery remains the primary treatment option, although the efficacy of adjuvant radiotherapy needs to be confirmed by further studies.

**Patient concerns:**

We report a rare case involving a 38-year-old woman who presented, in May 2022, with a large, borderline lobulated tumor in her left breast, measuring 35 cm × 30 cm. She needed to physically support the mass when performing any activity, and even slight physical activity elevated her heart rate to 130 beats/min. In addition, the patient was unable to lie flat and could only sleep on her left side.

**Diagnoses:**

Breast B-ultrasound examination and chest computed tomography scans showed the possibility of inflammatory changes. Ultrasound-guided pathologic examination of the mass could not determine the type of mass. Immunofluorescence and bacterial culture of the aspirated fluid were also negative, ruling out the possibility of infection. A mastectomy was then performed to clarify the diagnosis.

**Interventions:**

The tumor was completely removed, and the patient did not receive any adjuvant therapy after surgery.

**Outcomes:**

The patient recovered smoothly. Unfortunately, she experienced a recurrence of the left breast mass six months later, which progressed to malignancy.

**Lessons:**

The most effective treatment for phyllodes tumors of breast is wide local excision with clean margins greater than 1 cm. Simple mastectomy is recommended for borderline or malignant cases, especially when it is difficult to achieve reliable negative margins. Although adjuvant radiotherapy and chemotherapy after surgery are not generally recommended as first-line treatments, it raises the question of whether the recurrence could have been delayed if the patient had received postoperative radiation therapy.

## 1. Introduction

Giant phyllodes tumors are rare fibroepithelial neoplasms, accounting for less than 1% of all primary breast tumors. They are most commonly found in women aged between 45 and 50 years^[1]^ and are often unilateral. First described by Johannes Muller in 1838, giant borderline phyllodes tumors are defined as those with a diameter greater than 10 cm. They account for about 20% of all phyllodes tumors.^[2,3]^ In rare cases, these tumors can reach sizes of up to 30 cm in diameter. In this study, we present a rare case of a giant borderline phyllodes tumor measuring 30 cm in diameter and discuss the management strategies for patients with such large tumors. We also review the diagnostic and treatment options in light of the relevant literature.

## 2. Case presentation

**Figure 1.**
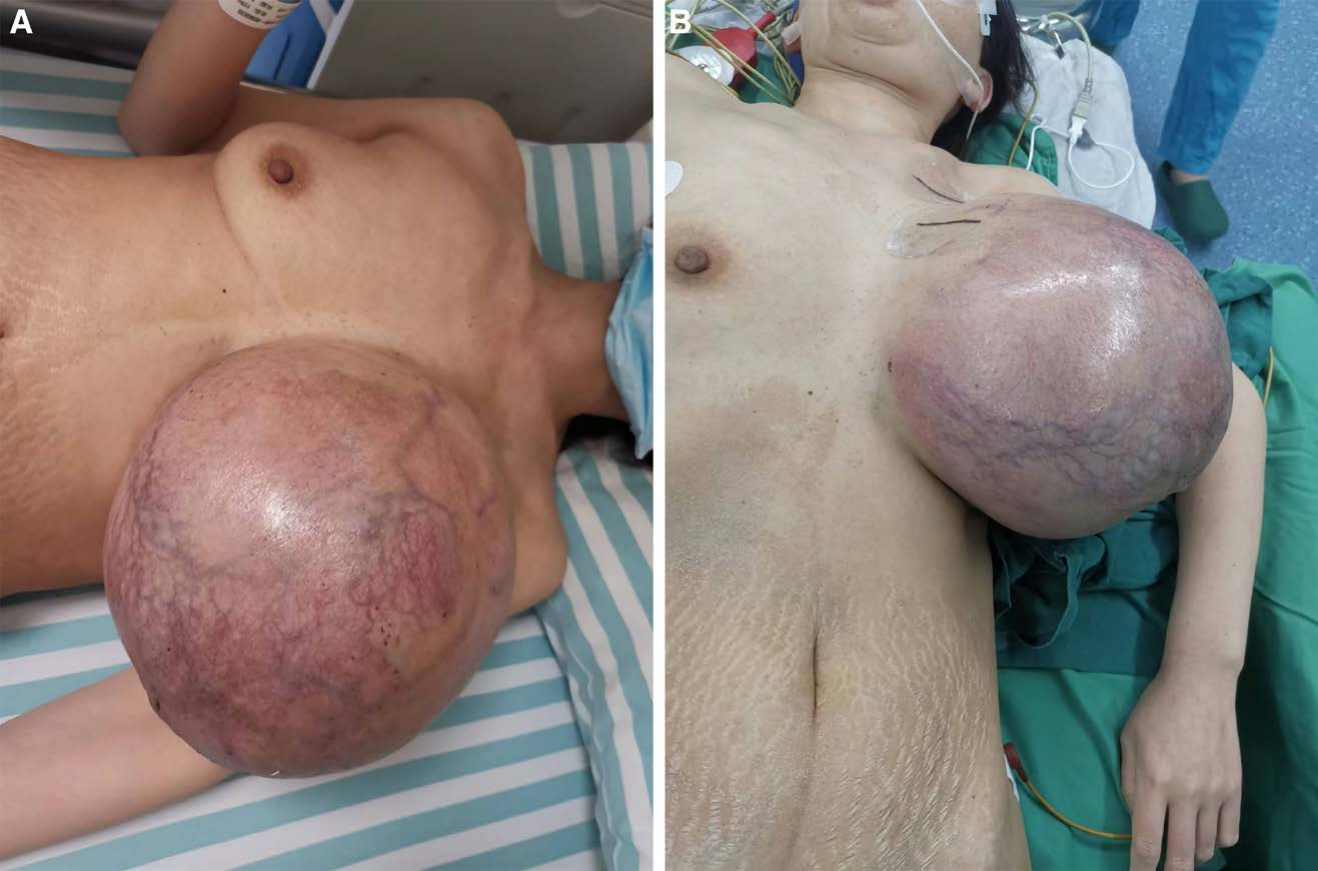
Patient’s left breast lump on admission (size approximately 35 cm × 30 cm).

A 38-year-old woman presented to another hospital with an egg-like mass in her left breast in March 2022. She reported no obvious cause or accompanying symptoms such as breast pain, nipple discharge, ulceration, redness, swelling, or fever. Initially, the patient had applied breast cream and herbal medicine (details unknown) but observed no significant improvement. Over 2 months, the mass progressively enlarged, which seriously affected the patient’s daily life. She needed to physically support the mass when performing any activity, and even slight physical activity elevated her heart rate to 130 beats/min. In addition, the patient was unable to lie flat and could only sleep on her left side. She visited our hospital on May 17, 2022. At this time, the mass was about 35 cm × 30 cm in size, roughly the size of a basketball (Fig. 1). The patient had no family history of tumors.

### 2.1. Physical examination

On visual examination, there was marked asymmetry between the 2 breasts, with the left breast showing a basketball-like mass measuring approximately 35 cm × 30 cm (Fig. 1). The shape of the left breast was distorted, with a smooth surface, visible vascularity, and nipple dimpling. The mass was firm to palpation and showed blurred borders. It was difficult to move and occupied the entire left breast. No enlarged lymph nodes were found in the axilla or the clavicle. No obvious abnormalities were detected in the head, neck, lungs, abdomen, or any other parts of the body.

### 2.2. Auxiliary examination

A comprehensive blood panel was conducted, including routine blood tests, liver and kidney function tests, and tumor markers. The following abnormal results were observed: hemoglobin: 89 g/L (reference range [RR]: 115–150 g/L), albumin: 31.8 g/L (RR: 35–55 g/L), total protein: 62 g/L (RR: 65–85 g/L), which showed that the patient had significant moderate anemia and hypoproteinemia.

**Figure 2.**
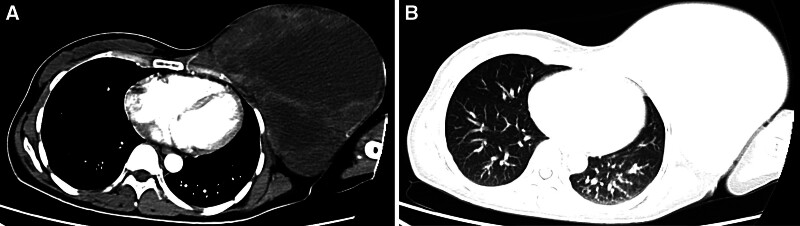
CT image shows a large, slightly dense mass with heterogeneous density in the left breast (A), with mild to moderate heterogeneous enhancement at the edge of the lesion on enhancement scan (B). CT = computed tomography.

Breast B-ultrasound examination showed abnormal echogenicity of the left breast, which, when combined with the patient’s medical history, suggested the possibility of inflammatory changes. Owing to limited tissue availability, ultrasound-guided mass aspiration biopsy also failed to provide a definitive determination of the type of pathology. No abnormal lymph nodes were detected via superficial lymph node ultrasound. Owing to the considerable size of the patient’s mass, her elevated heart rate, and her overall weakened condition, she was unable to complete a breast magnetic resonance imaging (MRI). Consequently, a chest computed tomography (CT) scan was performed (Fig. 2), which revealed a huge mass in the left breast, likely to be a borderline mass of the interlobular breast, not excluding infectious lesions. No abnormalities such as metastatic signs were seen on abdominal CT.

### 2.3. Admission diagnosis

At the time of admission, the left breast mass of the patient was enormous, with a diameter of 30 cm, and had rapidly increased in size within 2 months. Owing to the huge size of the mass and the patient’s limited mobility, she was unable to undergo mammography and MRI. Only ultrasound and chest CT scans could be performed, both of which suggested the possibility of an infection. Subsequently, we performed an ultrasound-guided aspiration of the mass for pathological examination and extracted 45 mL of reddish-brown bloody fluid. The type of mass could not be identified based on the aspirate. Meanwhile, immunofluorescence examination and bacterial culture of the extracted fluid returned negative results, ruling out the possibility of infection. Superficial lymph nodes examined via ultrasound and abdominal CT scans also showed no signs of metastases. To clarify the type of mass and establish an appropriate treatment plan, we decided to perform a left-sided mastectomy for paraffin pathological examination. Before the surgery, an ultrasound was conducted to locate the vascular pathways of the breast; this helped to determine that the blood supply of the tumor originated from the penetrating branches of the intrathoracic vessels, as well as from the lateral thoracic and thoracodorsal arteries. Identifying these branches and pathways was important to facilitate intraoperative hemostasis, and to prepare blood for the operation to manage any potential complications.

### 2.4. Process of surgical treatment

**Figure 3.**
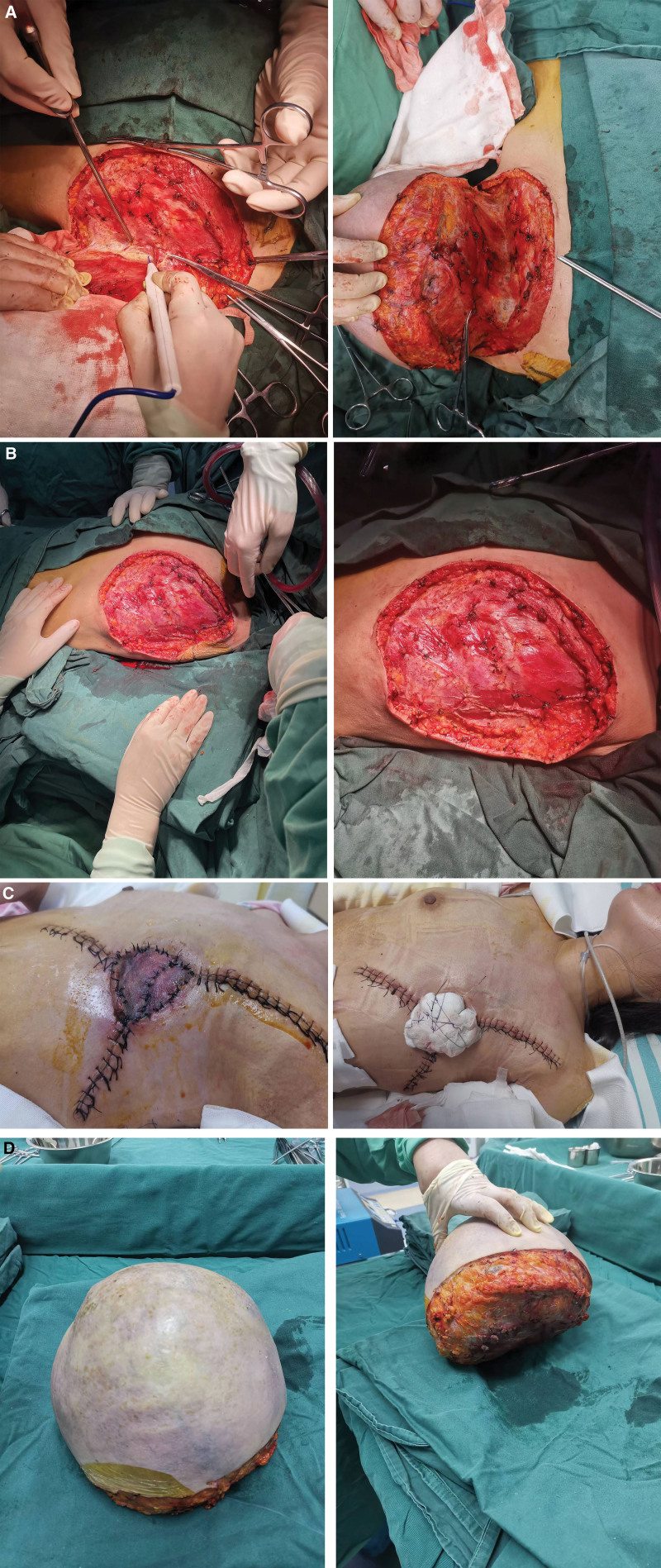
The steps of the surgical procedure. The mass was carefully separated during surgery (A); a large skin defect was visible in the excised mass (B); the defect was subsequently successfully repaired (C); and the mass weighed 4.5 kg after surgery (D).

As shown in Figure 3A, the patient underwent a surgical resection of the huge left breast tumor on June 1, 2022. After the onset of anesthesia, routine disinfection, draping, and hand-wrapping procedures were carried out. A circular incision was made along the edge of the giant left breast tumor. Using a high-frequency electric knife, the skin and subcutaneous tissues were incised up to the clavicle, to a depth of 2 cm at the midline, extending laterally along the sternal interruption and outside to the anterior axillary line. The tumor and its surface skin were carefully freed from the surface of the chest wall. Adhesions were observed between the base of the tumor and the pectoral muscles. After resection, a huge defect measuring approximately 35 cm × 30 cm was left in the left chest wall of the patient (Fig. 3B), which was successfully repaired by combining arbitrary flap plasty and full-thickness autologous skin graft with a subcutaneous vascular network (Fig. 3C). The excised mass (Fig. 3D) weighed about 4.5 kg and was immediately sent for routine pathological biopsy along with tissue samples from the surgical margins. Intraoperative bleeding was well controlled, amounting to 700 mL, and was addressed with 4 units of blood transfusion. The patient recovered smoothly from postoperative anesthesia and safely returned to the ward. The skin graft showed excellent survival, without any intraoperative or postoperative complications.

### 2.5. Postoperative pathological diagnosis

**Figure 4.**
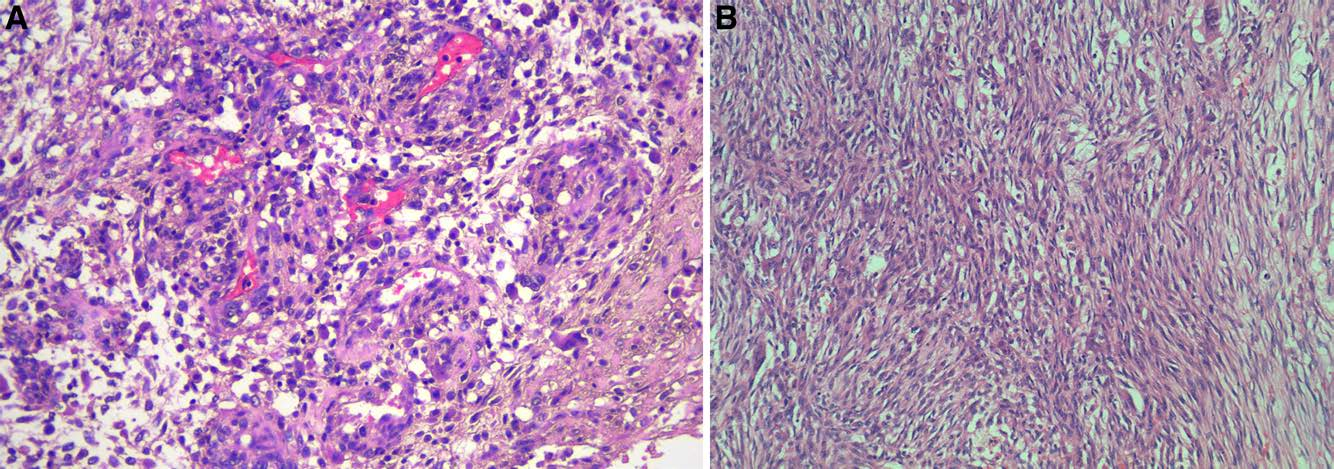
Postoperative pathology, consistent with a borderline lobular tumor.

Routine postoperative pathological biopsy of the tumor revealed the following characteristics: The borders of the tumor tissue were indistinct, and the focal points showed infiltrative growth. Cells were arranged in bundles that were unevenly spaced. The cells had a medium cytoplasm with distinct nucleoli, and nuclear division images were visible in the cells, with a count of about 4 to 8 per 10 high-power fields. Fissure-like breast ducts and numerous thick-walled vessels were observed between the karyograms, along with extensive hemorrhagic necrosis. These features were consistent with a diagnosis of a borderline lobulated tumor (Fig. 4). Postoperative examination confirmed that the surgical margins of the mass were negative.

### 2.6. Postoperative treatment

Currently, it is generally accepted that adjuvant radiotherapy and chemotherapy are not significantly effective in treating lobular tumors. In addition, the patient’s financial constraints precluded the use of such therapies. Therefore, the patient did not receive any postoperative adjuvant therapy.

**Figure 5.**
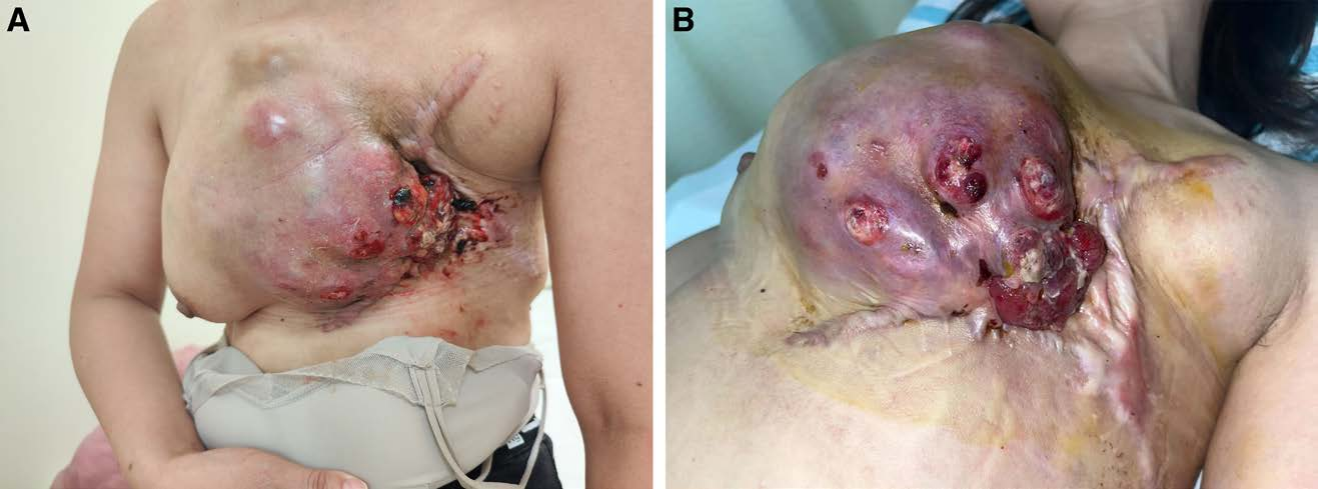
Recurrence of the mass in the left breast 6 mo later, which progressed to malignancy.

**Figure 6.**
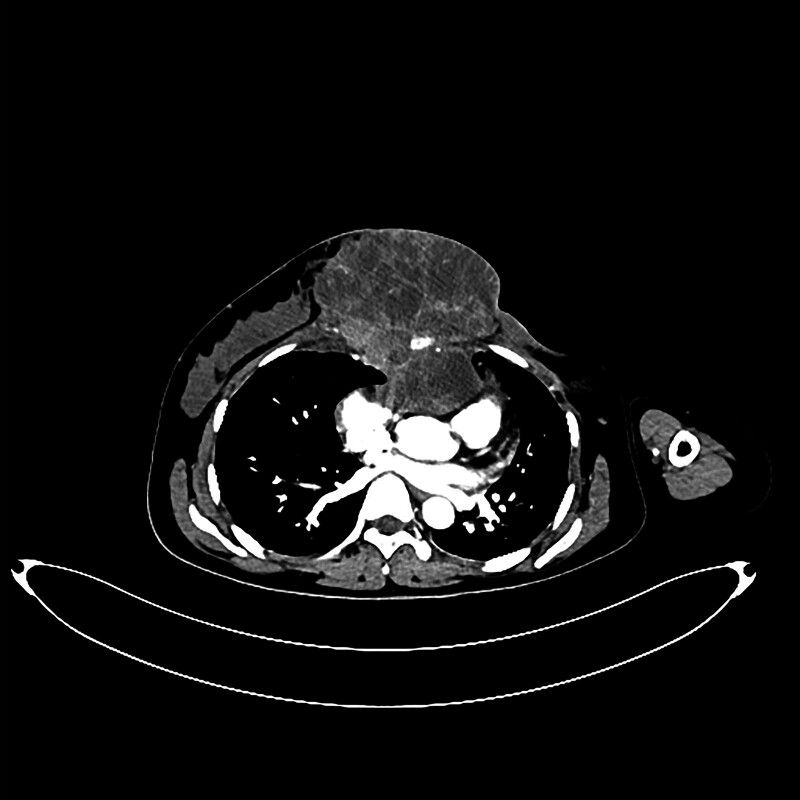
CT image shows postoperative changes in the left breast, alongside a large soft tissue mass in the anterior and middle chest wall. The mass invades the sternum and anterior mediastinum. CT = computed tomography.

Unfortunately, the patient experienced a recurrence in January 2023 and returned to our hospital in June 2023. Upon examination, a huge mass (Fig. 5) was observed on the left side of her chest wall, which was adherent and fixed to the chest wall, rendering it immovable. The surface of the mass showed ulceration and pus. Enlarged lymph nodes were palpable in the left axillary region. Chest CT scans (Fig. 6) suggested that the recurrent tumor had invaded the sternum and anterior mediastinum. Multiple metastatic nodules were also observed in both lungs. Eventually, the patient refused a tissue biopsy of the mass and opted to seek treatment at a higher-level hospital.

## 3. Discussion

Phyllodes tumors are rare fibroepithelial breast tumors that have both epithelial and stromal elements and account for less than 1% of all breast tumors. These tumors mostly affect women aged between 45 and 49 years.^[1]^ They are often unilateral in origin, with a less than 1% chance of bilateral occurrence at the same time.^[4,5]^ Although instances of phyllodes tumors have also been reported in men,^[6]^ they are extremely rare. Based on the number of mesenchymal cells, cell heterogeneity, nuclear division, tumor margins, and the presence or absence of stroma, the World Health Organization classifies phyllodes tumors into 3 categories: benign, borderline, and malignant, with the majority being clinically benign.^[7]^

In clinical practice, lobular tumors are characterized by a single, mobile, round mass with painless, progressive enlargement, which is often the main reason for a medical consultation. However, there is no established correlation between the growth rate of the tumor and its potential for malignancy.^[8]^ While rapid growth and high volume could suggest the possibility of a lobular tumor, these characteristics do not reliably distinguish them from other benign breast conditions.

The clinical diagnosis of phyllodes tumors relies mainly on the histological examination after complete excision of the mass. In this study, after ruling out the possibility of inflammatory changes, our primary differential diagnoses were phyllodes tumor and giant fibroadenoma. It is necessary to distinguish between these 2 types of fibroepithelial tumors. Giant fibroadenomas mostly occur in young women. The tumor is slow-growing, enveloped, firm, and usually without nuclear atypia. In contrast, phyllodes tumors mostly occur in middle-aged and elderly women, show fast growth, have a softer texture, and commonly show nuclear atypia. Most imaging modalities such as ultrasound, mammography, and MRI often struggle to reliably distinguish between fibroadenomas and phyllodes tumors.^[9,10]^ Given the patient’s history and the results of ancillary examinations, we suspected a phyllodes tumor. Eventually, our suspicion was confirmed through complete surgical resection followed by postoperative histological examination (Fig. 4). Histologically, phyllodes tumors are characterized by their fibroepithelial composition and an irregularly expansile, cellular proliferation of specialized stroma. In general, unlike fibroadenomas, phyllodes tumors often show a prominent leaf-like architecture.^[11]^ Evaluation of stromal features is another important histological differentiator between phyllodes tumors and fibroadenomas. Phyllodes tumors generally have higher stromal cellularity density and significant stromal nuclear heterogeneity compared to fibroadenomas.^[11]^ Several studies have demonstrated that phyllode tumors could originate from fibroadenomas and that they share some molecular characteristics. However, the exact mechanism underlying this relationship remains unclear.^[12,13]^

The primary treatment for phyllodes tumors is surgical resection. However, they cannot be adequately treated by simple enucleation, which is commonly used in the treatment of fibroadenomas.^[14–16]^ Because of the high rate of local recurrence, the recommended approach for treating these tumors is a wide local excision that ensures a clear margin of at least 1 cm.^[3,16–18]^ It has been reported that benign, borderline, and malignant phyllodes tumors have recurrence rates of 10% to 17%, 14% to 25%, and 23% to 30%, respectively.^[19]^ An inadequate margin after excision is significantly associated with recurrence. However, recent studies suggest that the emphasis on a wide surgical margin might be less critical for benign phyllodes tumors than previously believed, according to a growing body of research.^[20–23]^ Therefore, larger studies are needed to confirm whether the traditional surgical approach involves “overtreatment” of benign lobular tumors. Simple mastectomy is recommended for borderline or malignant phyllodes tumors, especially when reliable negative margins are not available or when there are frequent local recurrences. In general, phyllodes tumors recur with a histology similar to the primary disease, but with the potential to transform into more aggressive tumors.^[16,18,24,25]^ It is clear that in this case report, the patient’s recurrent mass had progressed from borderline to malignant.

A meta-analysis concluded that tumor size, moderate to severe mesenchymal heterogeneity, mesenchymal cell overgrowth, tumor necrosis, and tumor border and margin status are independent prognostic factors for recurrence.^[26]^ Phyllodes tumors spread through hematogenous dissemination. The lungs, bone, and abdominal viscera are the most common sites of distant metastasis.^[8]^ Interestingly, the incidence of axillary lymph node metastasis is less than 5%. Consequently, axillary lymph node dissection is not necessary unless pathological examination confirms that the tumor has metastasized to the axilla.^[8]^ It has been shown that there is no significant difference in overall survival and metastasis-free periods between local excision and mastectomy, although the recurrence rate associated with local excision is higher.^[27]^ In this study, as the patient’s tumor had spread to the entire breast, we removed the entire breast and ensured that the surgical margins were clear of any tumor presence, during the initial treatment. We also took utmost care to preserve the patient’s pectoralis major and pectoralis minor muscles.

In terms of postoperative care for phyllodes tumors, there is currently some controversy regarding adjuvant radiation therapy. In a study, Zeng et al^[28]^ observed that in patients with borderline or malignant lobular tumors, especially those who underwent local mastectomy, postoperative adjuvant radiation therapy improved survival and reduced local recurrence rates. In contrast, in a clinical study on 77 patients, Ossa et al^[29]^ showed that adjuvant radiation therapy had no effect on patient survival. Another study mainly focusing on benign lobular tumors also showed no effect of radiation therapy on local recurrence.^[30,31]^ A systematic review and meta-analysis conducted by Chao et al^[32]^ revealed the potential benefits of radiation therapy in patients aged below 45 years, with tumors measuring more than 5 cm, and those diagnosed with malignant tumors. Some experts have suggested that in patients with non-negative surgical margins, adjuvant radiation doses ranging from 50 to 60 Gy may be considered to reduce the risk of local recurrence.^[16,17]^ Although the use of adjuvant radiotherapy in reducing local recurrence rates has increased in recent years,^[33,34]^ limited data from large prospective studies are available to support the benefits of radiation therapy for phyllodes tumors.

In a prospective study, it was observed that chemotherapy offers negligible benefits in terms of survival.^[35]^ However, in one report, a combination of doxorubicin and cisplatin demonstrated complete remission and good palliation,^[36]^ while another study reported that a combination of etoposide and cisplatin demonstrated antitumor activity.^[37]^ Another study concluded that alkylating-agent-based chemotherapy regimens are effective in treating unresectable metastatic phyllodes tumors.^[38]^

In conclusion, chemotherapy is not recommended as the first-line treatment for phyllodes tumors thus far. However, in extreme cases, such as when the tumor size is extremely large or when adjacent structures, such as the chest wall, are involved, the use of chemotherapy may be considered.^[39]^

The role of hormone therapy in the treatment of phyllodes tumors remains unclear.^[12,40]^ It is believed that phyllodes tumors have estrogen and progesterone receptors on their epithelial surfaces, and the metastatic spread of these tumors is related to their mesenchymal components.^[40]^

When the patient was admitted to the hospital, her tumor was the size of a basketball. After adequate preoperative preparation, the mass was successfully removed, ensuring clear margins. The patient recovered well and was subsequently discharged from the hospital. However, 6 months later, the tumor in the patient’s left breast recurred and progressed to malignancy. After reviewing the entire course of treatment and considering previous literature, we wondered whether the recurrence could have been delayed if we had resected the pectoralis major and minor muscles during surgery and followed up with postoperative radiation. In addition, timely visits by the patient might have enabled more rapid control of the condition. It is noteworthy that in today’s society, there remains a significant gap between the rich and the poor, and many individuals have a limited awareness of their health.

In conclusion, the diagnosis and treatment of phyllodes tumors require continuous learning and exploration. While surgical resection remains the traditional treatment for phyllodes tumors, addressing their high recurrence rate remains a challenge for surgeons. Large-scale prospective studies are needed to answer this question. The role of adjuvant treatment in managing phyllodes tumors remains controversial. With comprehensive data research, it is hoped that a more effective treatment strategy for phyllodes tumors will emerge in the future.

## Author contributions

**Writing – original draft:** Gongyin Zhang.

**Writing – review & editing:** Gongyin Zhang, Jinsheng Zeng, Changlong Wei.

**Supervision:** Changwang Li.
